# Linking geographic flavor signatures to microbial origin in high-temperature Daqu: An integrated metaproteomics and metabolomics approach

**DOI:** 10.1016/j.fochx.2026.103952

**Published:** 2026-05-06

**Authors:** Dandan Song, Xian Zhong, Guihu Zhang, Juan Chen, Yansong Xue, Liang Yang

**Affiliations:** a School of Brewing Engineering, Moutai Institute, Renhuai 564501, China; b School of Food Engineering, Moutai Institute, Renhuai 564501, China.; c Key Laboratory of Food Bioengineering (China National Light Industry), College of Food Science and Nutritional Engineering, China Agricultural University, Beijing, China

**Keywords:** Enzymatic mechanism, Flavor formation, Functional decoupling, High-temperature Daqu, Metaproteomics, Microbial terroir

## Abstract

Elucidating the molecular architecture of microbial terroir is vital for precision fermentation, yet functional decoupling between taxonomic abundance and in situ expression remains a fundamental challenge. To resolve this “abundance-activity paradox,” we integrated metaproteomics, metabolomics, and metagenomics across the Chishui River gradient. We identified distinct chemosensory fingerprints: upstream thermotolerant consortia (*Bacillus* and *Oceanibacillus*) specialize in 2,3,5,6-tetramethylpyrazine biosynthesis mediated by bacterial acetolactate decarboxylase, while downstream microbiota (*Weissella* and *Debaryomyces*) prioritize alcohol and ester formation. Crucially, metaproteomic profiling unmasked the “rare biosphere” as a primary driver of core metabolic fluxes. While *Bacillus* was genomically dominant, keystone functional taxa—specifically low-abundance fungi like *Hyphopichia* and *Paecilomyces*—were the actual executors of rate-limiting starch hydrolysis. Furthermore, functional resilience was uniquely maintained through robust fungal co-occurrence networks despite geographic constraints. This study challenges abundance-centric paradigms, providing an activity-based framework for the rational design of synthetic microbial consortia to standardize flavor while preserving regional identity.

## Highlights

• Strong distance-decay in taxonomy, yet functional resilience via fungal networks.
• Functional niche partitioning along river: pyrazines upstream, alcohols downstream.
• An activity-based roadmap for rational design of fermentation starters.
• Active minorities, not dominant taxa, drive geographic flavor signatures.
• Metaproteomics identifies key microbial drivers of Daqu flavor formation.

## Graphical abstract

**Figure:**
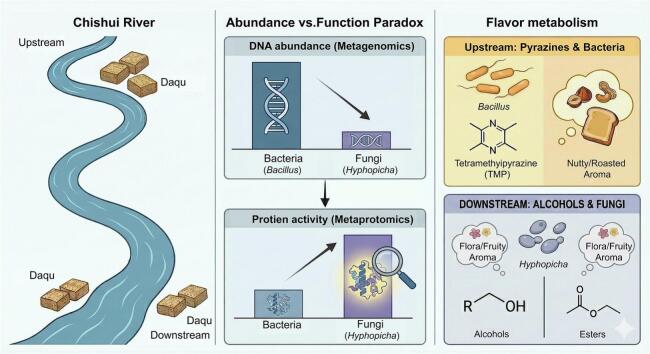

## 1 Introduction

Within food engineering systems such as fermented foods, microbial communities function as “biological catalysts” that drive critical biochemical transformations governing product quality and safety. Baijiu production, for instance, fundamentally depends on multi-strain solid-state fermentation, wherein the quality and distinctive sensory attributes predominantly rest on the diversity and stability of resident microbiota. As the primary fermentation starter for Jiang-flavor baijiu, *high-temperature Daqu* serves as a microbial repository governing fermentation kinetics and metabolic outputs (Bresciani et al., 2025; Niu et al., 2025). When uniformly blended with sorghum at a 1:1 ratio, *Daqu* serves as the fundamental reservoir of microbial consortia, enzyme systems, and critical metabolic precursors for Moutai-flavor Baijiu production. This intricate microbiome executes starch saccharification, proteolysis, and thermophilic fermentation (60–65 °C), yielding signature flavor compounds dominated by pyrazines and esters (Hou et al., 2024; Luo, Liao, Wu, et al., 2025; Zhu, Chen, et al., 2023). Divergent regional microbiomes uniquely shape local Baijiu styles, positioning high-temperature *Daqu* as an optimal model for deciphering Baijiu's geographical signatures. Firstly, within the Chishui River Basin—the epicenter of Moutai-flavor Baijiu production—spatial heterogeneity in abiotic factors drives selection-for-function in microbial taxa, establishing the liquor's microbial terroir. Secondly, assembly mechanisms governing microbial community architecture govern metabolic resilience, with endemic strains contributing to region-specific flavor profiles. Thirdly, delineating conserved “core microbiomes” across production systems delivers dual benefits: enabling standardized *Daqu* production while safeguarding inherent sensory attributes of traditional processing.

Existing studies reveal distinct variations in microbial community structures of high-temperature *Daqu* across geographical regions. In Hubei, *Kroppenstedtia* dominates among bacteria while *Aspergillus* prevails among fungi (Hou et al., 2024). Guizhou samples additionally host *Virgibacillus*, *Oceanibacillus* as core microbiota (Yang, Fan, & Xu, 2024a). Notably, Sichuan-derived *Daqu* exhibits significantly higher abundance of *Saccharopolyspora* (Deng et al., 2020). These findings demonstrate pronounced biogeographical isolation governing high-temperature *Daqu* fermentation, likely driving region-specific divergence in qu-aroma compound synthesis that ultimately shapes baijiu sensory characteristics. While amplicon sequencing (16S/ITS) has characterized dominant taxa including *Bacillus*, *Thermoactinomyces*, *Aspergillus*, and *Thermoascus* (Niu et al., 2025; Y. Wang et al., 2021; Yang, Fan, & Xu, 2024a; Zhang, Tan, et al., 2022), functional predictions based primarily on taxonomic inference overlook gene expression levels, limiting the study of key functional microbes. The abundance of uncultivable or fastidious microorganisms in *Daqu* further impedes mechanistic validation (Zhang, Xu, et al., 2022). While metagenomics offers advantages over amplicon sequencing for functional gene annotation and resolution of core fermentation pathways (Hou et al., 2024; Leech et al., 2020; N. Li et al., 2023; J. Xu, 2024), yet it reveals only the microbiome's genetic potential (i.e., putative function). it is often limited by the assumption that numerically dominant taxa drive community function and flavor formation. In stressful environments like high-temperature *Daqu* fermentation, DNA-based taxonomic abundance can largely decouple from actual metabolic activity, and may sporulate in response to environmental fluctuations, contributing abundant genomic DNA but exhibiting negligible enzymatic activity (Xiaolong Hu et al., 2020). Conversely, low-abundance microorganisms can be highly active and govern rate-limiting metabolic fluxes. Relying solely on metagenomic sequencing only reveals the microbiome's genetic potential (i.e., putative function), obscuring the actual functional executors.

To address this limitation, integrating metaproteomics allows for the direct quantification of expressed enzyme systems (i.e., realized function), overcoming the predictive constraints of metagenomic analyses (Van Den Bossche et al., 2025). This approach shifts the research focus from taxonomic abundance to metabolic activity, redefining keystone species by their real-time catalytic contributions rather than their population size. Consequently, associating regional phenotypic variations in high-temperature *Daqu* with proteomic signatures allows for precise identification of core functional microbiota. This research addresses a significant knowledge gap in brewing microbiology by elucidating the geographical provenance of *Daqu*'s characteristic flavor compounds and their linkage to functional microbial communities. Through an integrated multi-omics strategy combining metaproteomics, metabolomics, and metagenomics, we investigated the phenotypic differentiation of high-temperature *Daqu* across various production areas within the Chishui River Basin, and establishing a ternary framework that links microbial community, function, and flavor. Crucially, we hypothesize that the true drivers of geographic flavor specificity may not be the most abundant taxa, but rather low-abundance microorganisms with high metabolic activity.

## 2 Materials and methods

### 2.1 Daqu sampling

High-temperature *Daqu* samples were collected from six Maotai-flavor Baijiu factories in the upper, middle, and lower reaches of the Chishui River. Collected samples were snap-frozen in liquid nitrogen, milled into powder, transferred into sterile bags, and stored at −80 °C.

### 2.2 Determination of enzyme activity

Crude enzyme extracts from *Daqu* (HTD) were prepared by homogenizing the sample with a buffer solution (Na₂HPO₄/C₆H₈O₇, pH 6.0) at a 1:10 (*w*/*v*) ratio for enzymatic assays. Glucoamylase and amylase activities were determined using previously described methods (5). One unit (U) of amylase activity was defined as the amount of enzyme liberating 1 mg of glucose within 5 min, whereas one unit (U) of glucoamylase activity was defined as the amount of enzyme releasing 1 mg of glucose in 30 min. Moisture content of HTD was calculated using the following equation: *Moisture content (%)* = [(wet weight – dry weight) / wet weight] × 100 (Zhang, Luo, et al., 2025).

### 2.3 Analysis of non-volatile metabolites

#### 2.3.1 Sample preparation and extraction

The *Daqu* sample stored at −80 °C refrigerator was thawed on ice. A 400 μL solution (Methanol:Water = 7:3, *V*/V) containing internal standard was added into 20 mg sample, and vortexed for 3 min. The sample was sonicated in an ice bath for 10 min and vortexed for 1 min, and then placed at −20 °C for 30 min. The sample was then centrifuged at 12000 rpm for 10 min (4 °C). And the sediment was removed, then centrifuged the supernatant at 12000 rpm for 3 min (4 °C). A 200 μL aliquots of supernatant were transferred for LC-MS analysis.

#### 2.3.2 HPLC conditions

All samples were analyzed using two LC-MS/MS methods. One aliquot was analyzed using positive ion conditions and was eluted from T3 column (Waters ACQUITY Premier HSS T3 Column 1.8 μm, 2.1 mm × 100 mm) using 0.1% formic acid in water as solvent A and 0.1% formic acid in acetonitrile as solvent B in the following gradient: 5 to 20% in 2 min, increased to 60% in the following 3 mins, increased to 99% in 1 min and held for 1.5 min, then come back to 5% mobile phase B within 0.1 min, held for 2.4 min. The analytical conditions were as follows, column temperature, 40 °C; flow rate, 0.4 mL/min; injection volume, 4 μL; Another aliquot was using negative ion conditions and was the same as the elution gradient of positive mode. All the methods alternated between full scan MS and data dependent MSn scans using dynamic exclusion. MS analyses were carried out using electrospray ionization in the positive ion mode and negative ion mode using full scan analysis over *m*/*z* 75–1000 at 35000 resolution. Additional MS settings are: ion spray voltage, 3.5 KV or 3.2 KV in positive or negative modes, respectively; Sheath gas (Arb), 30; Aux gas, 5; Ion transfer tube temperature, 320 °C; Vaporizer temperature, 300 °C; Collision energy, 30,40,50 V; Signal Intensity Threshold, 1.00E+06 cps; Top N vs Top speed, 10; Exclusion duration, 3 s. Non-volatile metabolites analyzed via LC-MS/MS were annotated based on accurate mass and MS/MS fragmentation patterns matching with internal databases (MSI Level 2).

### 2.4 Analysis of volatile compounds

A precisely weighed 2.00 g aliquot of each ground sample was placed into a 20 mL sample vial, followed by the addition of 5 mL of deionized water and 1.8 g of sodium chloride (S. Yang et al., 2022). The volatile compounds were further evaluated by adopting GC–MS (7890B GC System, 5977 A MSD). Each sample (1 μL) was employed for analysis on a DB-FFAP capillary column (60 m × 250 μm × 0.25 μm, Agilent Technologies, Santa Clara, CA, USA). Helium (99.999%) was used as a carrier gas at a constant flow rate of 2.0 mL/min, and the inlet temperature was 250 °C. The oven temperature was maintained at 40 °C initially and was held for 3 min, ramped to 150 °C at a rate of 2 °C/min and was held for 2 min, and then finally was raised to 230 °C at a rate of 7 °C/min and held was for 8 min. The temperature of the transfer line was 250 °C, and that of the MS ion source was set at 230 °C. The ionization energy of the electron impact mass spectra was 70 eV, and the acquisitions were over an *m*/*z* scan range of 35–450 amu. The injection was performed in splitless mode. Three parallel experiments were conducted for each sample, and the volatile compounds were identified by comparing their mass spectra in the NIST (2020) baijiu flavor compounds database developed by our group and their RI from the DB-FFAP column with those of the authentic standards. The retention indices of the compounds were calculated using alkanes (C8–C40). The n-alkanes of C8–C40 were configured in the n-hexane solution, which was selected to take into account the protection of the column. The solution concentration was 20 μg/L. The same heating procedure as the sample was selected. The injection mode was splitless, and the injection volume was 1 μL. After the RI were calculated according to the formula, they were compared with the values in the literature so as to realize the further characterization of the compounds.

### 2.5 DNA extraction and metagenomic sequencing

0.2 g of *Daqu* was used to extract total genomic DNA with the FastPure Stool DNA Isolation Kit(Magnetic bead) (MJYH, Shanghai, China) according to manufacturer's instructions. Concentration and purity of extracted DNA was determined with SynergyHTX and NanoDrop2000, respectively. DNA quality was checked on 1% agarose gel. DNA extract was fragmented to an average size of about 350 bp using Covaris M220 (Gene Company Limited, China) for paired-end library construction. Paired-end library was constructed using NEXTFLEX Rapid DNA-Seq (Bioo Scientific, Austin, TX, USA). Paired-end sequencing was performed on Illumina NovaSeq™ X Plus (Illumina Inc., San Diego, CA, USA) at Majorbio Bio-Pharm Technology Co., Ltd. (Shanghai, China) using NovaSeq X Series 25B Reagent Kit according to the manufacturer's instructions (www.illumina.com).

The quality-filtered data were assembled using MEGAHIT (https://github.com/voutcn/megahit, version 1.1.2). Contigs with a length ≥ 300 bp were selected as the final assembling result. Open reading frames (ORFs) from each assembled contigs were predicted using Prodigal (Hyatt et al., 2010)(https://github.com/hyattpd/Prodigal,version2.6.3)and a length ≥ 100 bp ORFs were retrieved.

A non-redundant gene catalog was constructed using CD-HIT (Fu et al., 2012) (http://weizhongli-lab.org/cd-hit/,version 4.7) with 90% sequence identity and 90% coverage. Gene abundance for a certain sample was eatimated by SOAPaligner (R. Li et al., 2008) (https://github.com/ShujiaHuang/SOAPaligner,version soap2.21release) with 95% identity.

### 2.6 Taxonomic and functional annotation

The best-hit taxonomy of non-redundant genes was obtained by aligning them against the NCBI NR database by DIAMOND (Buchfink et al., 2015) (http://ab.inf.uni-tuebingen.de/software/diamond/,version 2.0.13) with an e-value cutoff of 1e-5. Similarly, the functional annotation (GO, KEGG, eggNOG, CAZy, CARD, PHI) of non-redundant genes was obtained. Based on the taxonomic and functional annotation and the abundance profile of non-redundant genes, the differential analysis was carried out at each taxonomic, functional, or gene-wise level by Kruskal-Wallis test.

### 2.7 Metaproteomics analysis

#### 2.7.1 Elution program

100 μg protein re-suspended with Triethylammonium bicarbonate buffer (TEAB) which with the final concentration of 100 mM. The mixture was reduced with Tris(2-carboxyethyl) phosphine (TCEP) which with the final concentration of 10 mM at 37 °C for 60 min and alkylated with iodoacetamide (IAM) which with the final concentration of 40 mM at room temperature for 40 min in darkness. After centrifugation at 10000 *g* at 4 °C for 20 min, the pellet was collected, which re-suspended with 100 μL Triethylammonium bicarbonate buffer (TEAB) which with the final concentration of 100 mM. Trypsin was added at 1:50 trypsin to-protein mass ratio and incubated at 37 °C overnight. The uPAC High Throughptu column (75 μm × 5.5 cm, Thermo, USA) was used with solvent A (water with 2% acetonitrile and 0.1% formic acid) and solvent B (water with 80% acetonitrile and 0.1% formic acid). The elution program was used as following gradient: 0–0.1 min, 4%–8% buffer B; 0.1–1 min, 8%–12.5% B; 1–1.1 min, 12.5%–12.6% B; 1.1–3.6 min, 12.6%–22.5% B; 3.6–5.8 min, 22.5%–45% B; 5.8–6.4 min, 45%–99% B; 6.4–8 min, 99%–99% B.

#### 2.7.2 DIA mass detection and protein identification

Data-independent acquisition data were acquired using an Orbitrap Astral mass spectrometer operated in DIA mode. The detection was carried out over a mass range of 70–1050 *m*/*z* (MS1), and 150–2000 m/z (MS2). The DIA mass spectrometric raw data were searched and processed with the Spectronaut software (Version 19) software. The parameters are as follows: the peptide length range was set to 7–52; Enzyme cutting site was trypsin/P; The maximum missed cleavage site was 2; Carbamidomethylation of cysteines as fixed modification, and oxidation of methionines and protein N-terminal acetylation as variable modifications; Protein FDR ≤ 0.01, Peptide FDR ≤ 0.01, Peptide Confidence ≥99%, XIC width ≤ 75 ppm. The protein quantification method was MaxLFQ.

### 2.8 Statistical analysis

The data were analyzed on the free online platform of Majorbio Cloud Platform (www.majorbio.com). *Daqu* sequences were analyzed on the online platform of Majorbio/I-Sanger Cloud Platform (https://cloud.majorbio.com/) (Han et al., 2024). Briefly, the raw sequencing reads were trimmed of adapters, and low-quality reads (length < 50 bp or with average quality value <20) were removed by fastp (https://github.com/OpenGene/fastp, version 0.20.0).Reads were aligned to the human genome by BWA (H. Li & Durbin, 2009) (http://bio-bwa.sourceforge.net, version 0.7.17) and any hit associated with the reads and their mated reads were removed. The microbe-metabolite co-occurrence network was constructed based on Spearman's rank correlation analysis. Only robust and statistically significant correlations (Spearman correlation coefficient |r| > 0.6 and *p* < 0.05) were retained as edges in the network visualization.

## 3 Results and discussion

### 3.1 Physicochemical and enzymatic characteristics

**Fig. 1:**
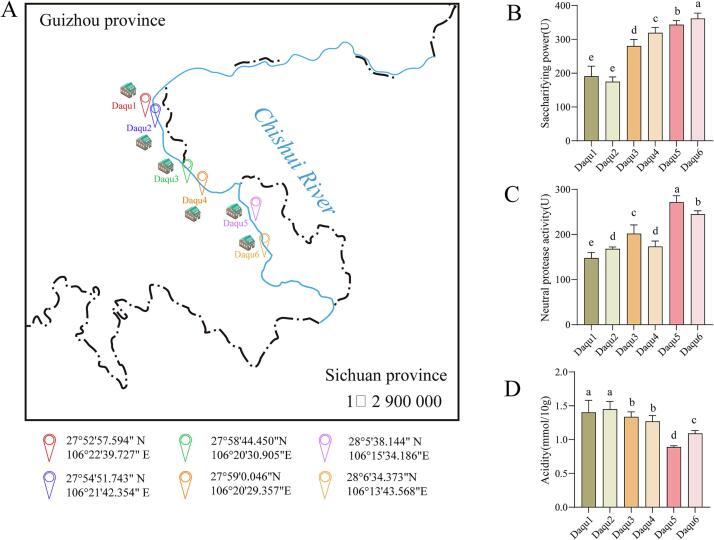
**Enzymatic activities and chemical characteristics of *Daqu* samples.** (A) Geographic coordinates and sampling locations of high-temperature *Daqu* along the Chishui River. (B) Saccharification power of *Daqu* across representative regions. (C) Neutral protease activity in regionally collected *Daqu*. (D) Acidity levels in *Daqu* samples from distinct regions.

Characterization of physicochemical parameters and key enzymatic activities is essential for assessing high-temperature *Daqu* quality and regulating fermentation dynamics (Wang, Wang, Huang, et al., 2020). We analyzed saccharifying power, protease activities, and acidity in *Daqu* samples collected from six production zones spanning the upper, middle, and lower reaches of the Chishui River (Fig. 1A). Downstream *Daqu* exhibited significantly higher amylase activity (*Daqu*6: 361.49 ± 13.33 U; *Daqu*5: 343.55 ± 9.67 U) compared to middle- and upstream counterparts (Fig. 1B). Neutral protease activities in downstream *Daqu* (271.63 ± 11.75 U and 244.78 ± 6.40 U) were markedly elevated relative to those from upper and middle reaches (Fig. 1C). Conversely, upstream *Daqu* samples displayed significantly greater acidity (1.40 ± 0.14 mmol/10 g and 1.45 ± 0.094 mmol/10 g) than those from middle and lower reaches (Fig. 1D). These distinctions are likely attributable to divergent raw materials, geographic conditions, microbial consortia, and metabolic functions (Yan et al., 2025; Zhang, Ran, et al., 2025). Overall, substantial variations in saccharification capacities persist among the three high-temperature *Daqu* types despite analogous production protocols, implicating differential microbial composition and metabolic outputs.

### 3.2 Comparative analysis of non-volatile metabolites in *Daqu* from various geographic origins

**Fig. 2:**
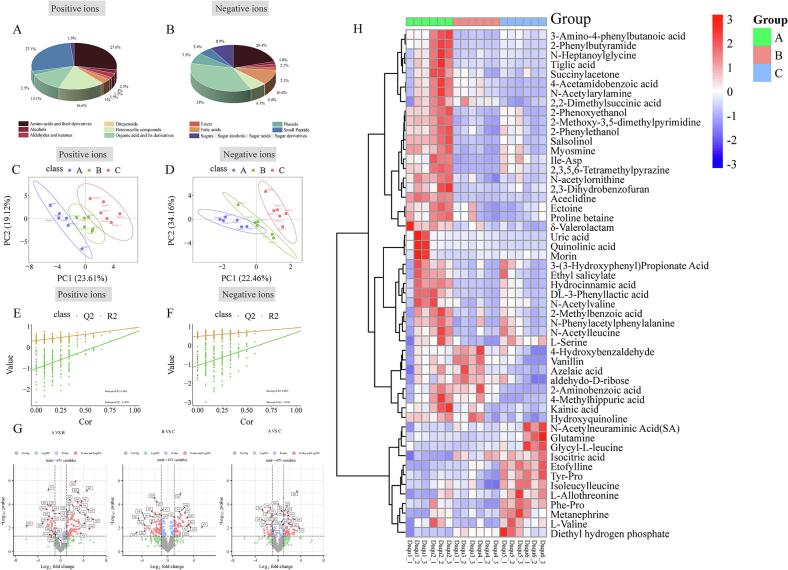
**Differences in non-volatile metabolites of *Daqu* from different river basins**. (A, B) Differences in compound categories and proportions under two detection modes. (C, D) PLS-DA analysis of *Daqu* compounds differences. (E, F) 200 permutation tests for PLS-DA. (G) Fold change comparisons of *Daqu* compounds across different basins. (H) Analysis of compound concentrations in *Daqu* from different basins.

Non-targeted metabolomics analysis of *Daqu* revealed 672 non-volatile metabolites detected in anion and cation modes. Cation-mode profiling was dominated by amino acids and derivatives (27.6%), small peptides (27.1%), heterocyclic compounds (16.6%), and organic acids/derivatives (13.1%) (Fig. 2A). Anion mode primarily identified phenolic compounds (35%), amino acids (20.4%), and sugar alcohol derivatives (8.9%) (Fig. 2B). PLS-DA clearly separated upstream (A), midstream (B), and downstream (C) groups along the Chishui River (Fig. 1C, D), with proximal clustering of A and B indicating metabolic similarity between upstream and midstream *Daqu*. Model validity was established through permutation testing (*n* = 200; Fig. 2E, F). Volcano plot analysis (Fig. 2G) identified 25 differential metabolites between A and B, substantially fewer than those between B and C (71 metabolites) or A and C (76 metabolites), highlighting distinct downstream metabolic signatures. Integration of volcano plot thresholds (*p* < 0.05, |log₂FC| >1; Table S1) and PLS-DA VIP scores (>1; Fig. 2H) defined watershed-specific markers. A-region *Daqu* showed significant enrichment of Ectoine, Proline betaine, 2,3,5,6-Tetramethylpyrazine, and 2-Phenylethanol—microbially derived secondary metabolites. Ectoine and Proline betaine, known as compatible solutes synthesized by halophilic/drought-tolerant bacteria for osmoregulation (Miao et al., 2025; Weinisch et al., 2018), likely support microbial viability in mature *Daqu* (∼10% moisture). Elevated levels of organic acids/derivatives (Vanillin, 4-Hydroxybenzaldehyde, d-Camphoric acid, 2-Aminobenzoic acid, Azelaic acid) in A and B may drive higher acidity in A and B. Conversely, C accumulated more amino acids (Glutamine, L-Allothreonine) and small peptides (Glycyl-*L*-phenylalanine, Ile-Lys), aligning with its enhanced protease activity and suggesting greater protease-producing microbial populations. Core flavor compounds (2,3,5,6-Tetramethylpyrazine and Vanillin in A) exhibited upstream enrichment. Notably, 2,3,5,6-Tetramethylpyrazine contributes nutty, roasted aromas to high-temperature *Daqu* and Moutai-flavor baijiu (Luo, Liao, Luo, et al., 2025). These watershed-dependent metabolic patterns reflect specialized microbial community structures and enzymatic networks, collectively governing flavor development and microbial adaptation strategies.

### 3.3 Geographic divergence in volatile compounds of *Daqu* across river reaches

**Fig. 3:**
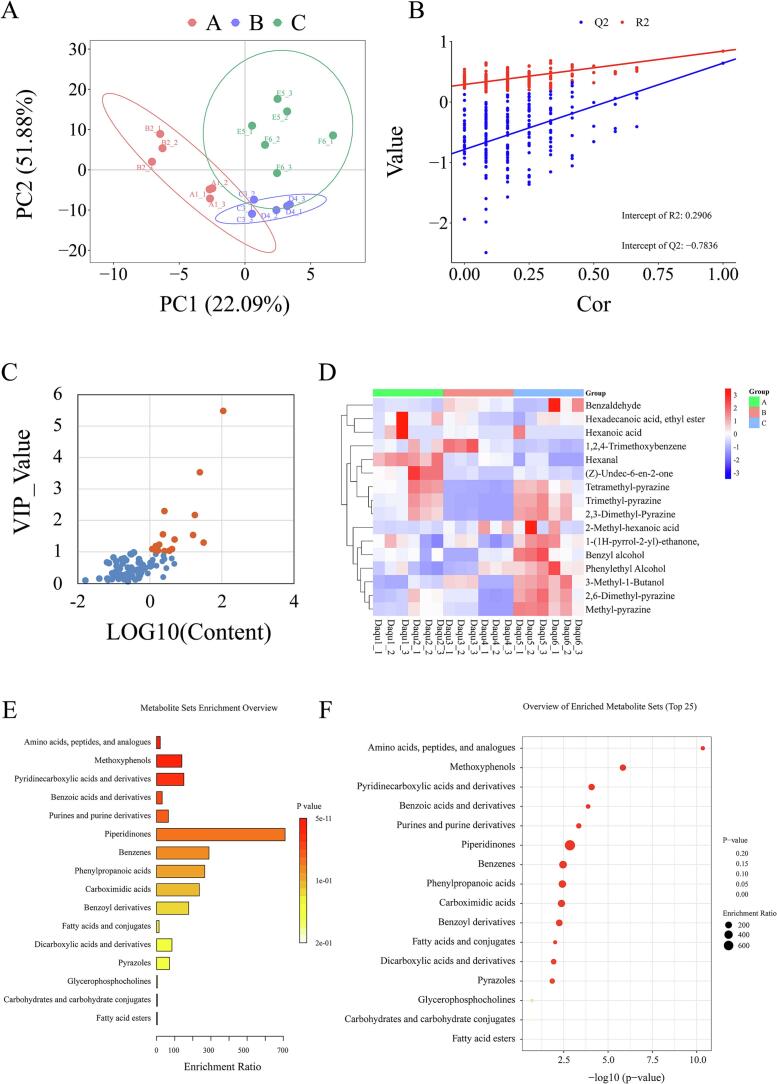
(A) PLS-DA of volatile compounds across *Daqu* variants. (B) Permutation testing. (C) VIP-Content score plot. (D) Abundance of differential metabolites among *Daqu* types. (E) KEGG enrichment analysis for non-volatile/volatile compounds. (F) Significance of pathway-based compound enrichment.

*Daqu* aroma significantly contributes to the flavor of Jiang-flavor baijiu. Therefore, the aroma of *Daqu* from different production areas influences the style of Jiang-flavor baijiu, making it essential to explore the specificity and core volatile compounds of *Daqu* across different river reaches. Using HS-SPME-GC–MS, we detected 83 flavor compounds in six types of high-temperature *Daqu*. PLS-DA successfully separated the volatile compounds of *Daqu* from three regions (Fig. 3A), indicating distinct flavor profiles among high-temperature *Daqu* from the three river reaches. This further confirms significant aroma differences in high-temperature *Daqu* across regions and suggests notable biogeographic characteristics in its microbial communities.

Sixteen characteristic compounds (VIP ≥ 1, concentration ≥ 10 μg/g) contributing most to regional differences were identified based on content and differential metrics (Fig. 3C). Comparative analysis of these volatile compounds (Fig. 3D) revealed that pyrazines, such as tetramethyl-pyrazine, trimethyl-pyrazine, and 2,3-dimethyl-pyrazine, were more abundant in upstream and downstream *Daqu*. Specifically, tetramethyl-pyrazine levels in upstream *Daqu* (169.35 ± 34.26 μg/g) were significantly higher than in midstream (7.72 ± 2.64 μg/g) and downstream *Daqu* (143.19 ± 49.5 μg/g), aligning with Non-targeted metabolomics' compound trends. Additionally, alcohols like benzyl alcohol, phenylethyl alcohol, and 3-methyl-1-butanol were elevated in downstream *Daqu*. The downstream 3-methyl-1-butanol content (2.71 ± 0.55 μg/g) significantly exceeded midstream (1.63 ± 0.78 μg/g) and upstream levels (1.15 ± 0.32 μg/g); phenylethyl alcohol reached 31.65 ± 6.21 μg/g, notably higher than upstream (19.48 ± 4.9 μg/g) and midstream *Daqu* (22.64 ± 5.67 μg/g).

Midstream *Daqu* exhibited higher levels of compounds such as 2-methyl-hexanoic acid and 1,2,4-trimethoxybenzene (Fig. 3D). These volatile disparities further demonstrate significant geographic differentiation in volatile metabolite composition among *Daqu* along the Chishui River. Pyrazines—known for nutty and roasted aromas—are typically produced by thermotolerant microorganisms (e.g., *Bacillus*) during high-temperature fermentation (He et al., 2025; Luo, Liao, Wu, et al., 2025). Alcohols like 3-methyl-1-butanol and phenylethyl alcohol derive from yeast-mediated Ehrlich pathway conversion of amino acids (Siripong et al., 2020), suggesting downstream conditions (e.g., lower temperature, higher humidity, or nutrient availability) favor yeast growth, enhancing alcohol fermentation and aroma synthesis.

Integrated enrichment analysis of volatile and non-volatile compounds (Fig. 3E) identified piperidinones, phenylpropanoic acids, and benzenes with high enrichment ratios, though amino acids, peptides, analogues, and methoxyphenols showed the most significant enrichment (*P* < 0.00001). As illustrated in Fig. 3F, amino acids, peptides, and analogues encompassed the most differentially abundant metabolites (*n* = 10), followed by methoxyphenols (*n* = 3) and benzoic acids/derivatives (*n* = 3), indicating pronounced disparities in nitrogen metabolism and aromatic compound synthesis among microbial communities across river reaches. Studies reveal that divergent amino acids (arginine, proline, glutamate, glutamine, tryptophan) correlate strongly with *Bacillus* (Xing et al., 2025), likely linked to its protease-producing capacity (Y. Liu et al., 2022; Zeng et al., 2022). This underscores *Bacillus*' critical role in nitrogen metabolism during high-temperature *Daqu* fermentation.Notably, methoxyphenols—particularly 2-methoxy-4-vinylphenol and vanillin—were markedly enriched in our study (Fig. 3F). These key aroma compounds (G. Shi et al., 2024) may arise from microbial ferulate decarboxylase activity (Langos & Granvogl, 2016; Witthuhn et al., 2012) Collectively, these results demonstrate marked geographical divergence in non-volatile and volatile metabolites in Chishui River *Daqu*, with compounds such as tetramethylpyrazine, 4-vinylguaiacol, phenethyl alcohol, and 3-methyl-1-butanol being most prominent. These differences likely stem from microbial community variations. Subsequent research should focus on enzymes and functional microorganisms linked to these differential compounds.

### 3.4 Geographical heterogeneity of microbial communities

**Fig. 4:**
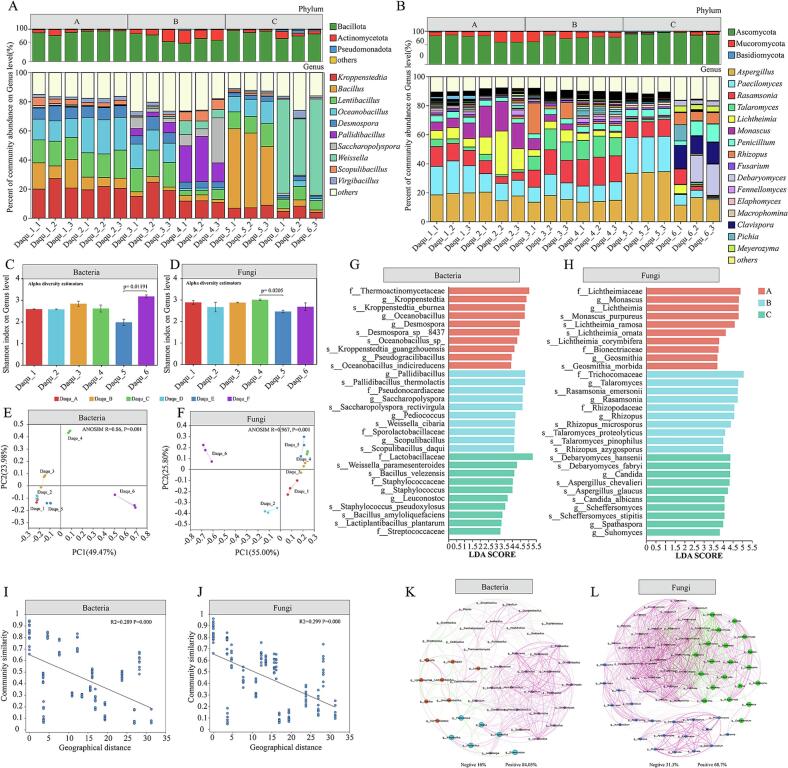
**Microbial community profiling of *Daqu* across distinct fermentation stages.** (A, B) Taxonomic composition of *Daqu* microbiota from sampled basins; (C, D) Alpha-diversity indices; (E, F) Non-metric multidimensional scaling (NMDS) ordination based on Bray-Curtis dissimilarity; (G, H) Linear discriminant analysis (LDA) effect sizes for fungal and bacterial taxa across basins; (I, J) Distance-decay relationships characterizing microbial community similarity; (*K*,*L*) Fungal-bacterial co-occurrence network patterns.

The microbiota involved in HTD fermentation primarily originates from natural enrichment of local environmental microorganisms and directional introduction of exogenous microbes, leading to significant differences in microbial composition and metabolic activity among different *Daqu* samples. Based on metagenomic analysis, we explored the microbial community structural variations in high-temperature *Daqu* across different river basins. We displayed the microbial composition of *Daqu* from distinct basins (Fig. 4A, B). Using RPKM values, we calculated the relative abundance of microorganisms in various *Daqu*. At the phylum level, predominant bacteria in high-temperature *Daqu* were Bacillota and Actinomycetota, with minor Pseudomonadota present in downstream *Daqu*; predominant fungi were Ascomycota and Mucoromycota, with minor Basidiomycota in downstream samples. At the genus level, high-temperature *Daqu* across all regions consisted mainly of bacteria such as *Kroppenstedtia*, *Bacillus*, *Lentibacillus*, *Oceanobacillus*, *Desmospora*, and *Scopulibacillus*; fungi included *Aspergillus*, *Paecilomyces*, *Rasamsonia*, *Talaromyces*, *Lichtheimia*, *Monascus*, and *Penicillium*. Unlike upstream *Daqu*, midstream *Daqu* contains more *Pallidibacillus* and *Saccharopolyspora*; downstream *Daqu* contains more *Weissella*. Significant differences are also observed in fungal communities. The mean relative abundance of *Monascus* in upstream regions is 7.3%, which sharply declines in midstream and downstream areas. *Talaromyces* shows lower abundance in downstream *Daqu*, while *Meyerozyma*, *Clavispora*, and *Debaryomyces* exhibit higher relative abundance. These results suggest distinct microbial community structures in *Daqu* across different river basins. Changes in microbial α-diversity of high-temperature *Daqu* were studied (Figs. 4C, D). The Shannon index shows no significant overall variation among six regions (Fig. 4C), indicating that species richness remains relatively stable across basins despite significant compositional differences. This stability likely results from selective pressures in the high-temperature fermentation environment maintaining the core microbiome. Non-metric multidimensional scaling (NMDS) revealed bacterial community clustering: *Daqu*1, *Daqu*2, *Daqu*3, and *Daqu*5 grouped together but differed significantly from *Daqu*4 and *Daqu*6 (ANOSIM *R* = 0.86, *P* = 0.001) (Fig. 4E). Fungal communities showed clear separation: *Daqu*1 and *Daqu*2 clustered distinctly from *Daqu*3 and *Daqu*4, while *Daqu*6 exhibited pronounced isolation (ANOSIM *R* = 0.967, P = 0.001).

To identify basin-specific microbial biomarkers, LEfSe analysis revealed: In watershed A, the LDA value of Thermoactinomycetaceae was 5.056, marking it as a significant biomarker. Key taxa also included Kroppenstedtia (4.91) and Desmospora (4.49), accompanied by *Oceanobacillus* (4.42) and Pseudogracilibacillus (3.92); Basin B was characterized by *Pallidibacillus* (4.53), *Saccharopolyspora* (4.51), and *Scopulibacillus* (4.13); downstream regions were dominated by *Lactobacillaceae* (5.25), primarily *Weissella* (4.25). *Bacillus* and *Staphylococcus* (4.07) were differential genera downstream, with *Bacillus* represented mainly by *B. velezensis* (4.15) and *B. amyloliquefaciens* (3.42). For fungi, upstream *Daqu* was distinguished by *Lichtheimiaceae* (mainly *Monascus* (4.73) and *Lichtheimia* (4.68)); Basin B was associated with *Trichocomaceae* (*Rasamsonia* (4.72), *Talaromyces* (4.68), *Rhizopus* (4.52)); Basin C was typified by *Weissella*, *Debaryomyces*, and *Aspergillus*. Studies reveal that microorganisms including *Saccharopolyspora*, *Pallidibacillus*, and *Scopulibacillus* exhibit thermotolerance up to 60 °C (Wu et al., 2016; Yao et al., 2016);In contrast, the upstream signature genus *Kroppenstedtia* survives temperatures ≤50 °C, while *Oceanobacillus* tolerates ≤45 °C (Nam et al., 2008);Downstream high-temperature *Daqu* is dominated by *Lactobacillaceae* with lower thermal resilience (Y. Sun et al., 2022),suggesting that microbial thermal adaptation drives community divergence across river basins—a variation attributable to distinct *Daqu*-production processes and environmental conditions (Yuan et al., 2025). Given that dispersal shapes community assembly (Wang, Wang, Shen, et al., 2020). we investigated spatial constraints on basin-specific microbiomes through distance-decay analysis (Fig. 4I, J). Similarity in bacterial communities of high-temperature *Daqu* declined significantly across geographical distances (R^2^ = 0.289; *p* < 0.001), paralleled by fungal communities (R^2^ = 0.299; p < 0.001). This distance-decay pattern confirms dispersal limitation in shaping *Daqu* microbiota composition, consistent with observations in freshwater bacterial assemblages, phyllosphere bacteria, and diverse ecosystems (Finkel et al., 2012; L. Liu et al., 2015). Co-occurrence network analysis further revealed that despite the distance-decay effect, specific microorganisms contribute to maintaining the stability of the high-temperature *Daqu* microbiome. Positive associations dominated both bacterial (84.05% of edges) and fungal networks, with fungal interactions being notably more extensive (1064 edges vs. 370 bacterial edges). Key bacterial genera exhibiting high positive connectivity—*Lentibacillus* (22 edges), *Oceanobacillus* (24 edges), and *Kroppenstedtia* (23 edges)—are likely ecological keystone taxa underpinning bacterial community stability. The fungal co-occurrence network largely comprised rare genera, suggesting their persistence may be facilitated by synergistic interactions(M. Zhu, Yin, et al., 2022). Crucially, *Paecilomyces* (43 edges) and *Penicillium* (42 edges), displaying the highest network connectivity, emerged as principal stabilizers of the fungal assemblage.

The present study demonstrates distinct microbial structures in high-temperature *Daqu* across different watersheds, suggesting these microbial biomarkers may correlate with variations in *Daqu*'s functional phenotypes. Furthermore, metagenomic analyses from multiple studies have characterized the microbial communities in high-temperature *Daqu*, identifying dominant bacterial genera such as *Bacillus*, *Kroppenstedtia*, *Oceanobacillus*, *Saccharopolyspora*, and *Staphylococcus*, along with key fungal genera including *Paecilomyces*, *Aspergillus*, *Rasamsonia*, *Thermomyces*, and *Thermoascus* (Luo, Liao, Wu, et al., 2025; Yang, Fan, & Xu, 2024b; Zhu, Cheng, et al., 2023), consistent with our findings. Moreover, the decay effect of geographic distance influences the similarity of microbial community composition in *Daqu* from different basins, which further verifies that the microbial community composition of high-temperature *Daqu* is constrained by dispersal processes. The divergence in microbial functionality—where upstream habitats select for pyrazine biosynthesis and downstream habitats favor alcohol/ester formation—reflects intense selection by long-term environmental pressures(G. Zhang et al., 2025). Upstream environments, with potentially lower ambient humidity and specific local micro-climates, create selective pressures that favor robust, thermotolerant r-strategists like *Bacillus*. Conversely, downstream facilities, characterized by higher environmental water activity and unique long-term domestication pools within the fermentation facilities, provide a niche highly conducive to the proliferation of fermentative yeasts and molds. However, significant microbial interactions sustain the basic microbial community of high-temperature *Daqu*, wherein its dominant microbial genera also function as crucial ecological keystones.

### 3.5 Region-specific heterogeneity in microbial community functionality

Metagenomic analysis revealed the compositional structure and functional potential of high-temperature *Daqu* microbial communities, identified key microbial taxa in samples from different watersheds, and predicted their potential metabolic pathways. These findings provide crucial insights for understanding the microbial basis of *Daqu* fermentation. However, metagenomics can only reflect “who is there” and “what they can do” but cannot directly confirm whether these genes are expressed and functionally active during actual fermentation. To elucidate the functional dynamics of the microbial community in *Daqu*, we employed proteomics to profile the expression of key enzymes and further investigated how microbiome functional expression contributes to the formation of *Daqu* with distinct functional attributes. We identified 35,610 polypeptides (Fig. S1A), with protein abundance peaking in the 11–20 kDa molecular weight range, followed by the 21–30, 31–40, and 41–50 kDa ranges (Fig. S1B). Functional annotation (GO/KEGG/COG) revealed that Molecular Function comprised the largest category, dominated by “catalytic activity” (20,918 proteins), signifying enzymatic predominance (Fig. S1C). COG annotation identified “amino acid transport and metabolism” as the most abundant functional category, succeeded by “carbohydrate transport and metabolism,” “energy production and conversion,” “lipid transport and metabolism,” and “general function prediction” (Fig. S1D), underscoring the central role of enzymes in defining *Daqu* functionality and flavor. Concordant KEGG analysis showed “carbohydrate metabolism” as the top pathway (5692 proteins), followed by “amino acid metabolism” (4176), “energy metabolism” (2896), and “translation” (2825) (Fig. S1E). These annotations consistently prioritized proteins integral to carbohydrate/amino acid metabolism and energy conversion, thereby pinpointing core functional microbiota governing *Daqu*'s metabolic phenotype. Research indicates that amino acid differences in *Daqu* serve as a crucial marker for evaluating its quality. Amino acids, particularly arginine, exhibit significant disparities between the two. Environments characterized by high temperature, high humidity, and low acidity contribute to the abundance of amino acids in *Daqu*, while mechanical pressing affects the stability of the core microbiota. The presence of amino acids is considered a novel potential biomarker influencing *Daqu* quality. (Xing et al., 2025).

**Fig. 5:**
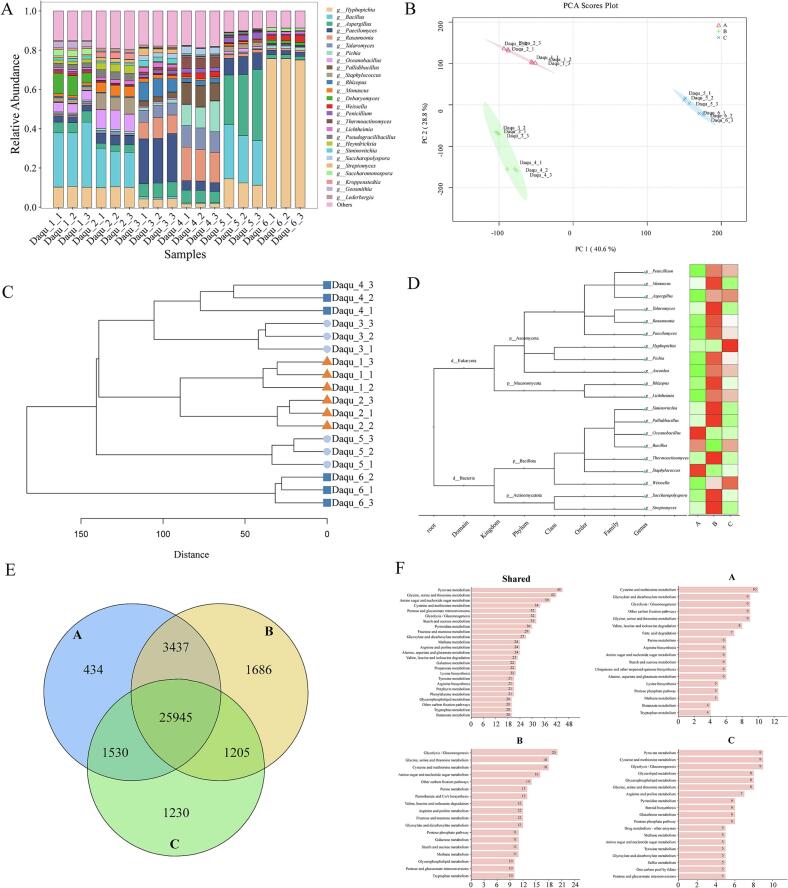
**Microbial community analysis across sample groups.** (A) Stacked bar plot showing relative abundances of microbial taxa, with colors indicating genera. (B) Principal coordinates analysis (PCoA) ordination based on Bray-Curtis dissimilarity, depicting structural divergence among groups (color-coded). (C) Hierarchical clustering analysis of samples using protein profiles. (D) Heatmap displaying microbial abundances annotated at the protein level, with rows representing taxonomic units and columns denoting samples. (E) Unique and shared protein sets identified in *Daqu* samples across watersheds. (F) Distribution frequency of unique and shared proteins in KEGG pathways.

Using metagenomic sequencing data, NR-based taxonomic annotation of detected proteins was performed. Species abundances were derived by aggregating proteins mapped to each taxon, generating taxonomically resolved abundance profiles (Fig. 5A). Fungal proteins dominated the proteome across *Daqu* from three geographically distinct regions, with the genus *Trichosporon* exhibiting the highest mean relative abundance (24%), followed by *Bacillus* (9.3%), *Paecilomyces* (6.9%), *Oceanobacillus* (5.2%), *Rasamsonia* (5.04%), and *Aspergillus* (4.5%). Significant spatial heterogeneity in microbial protein abundance was observed across production regions. Studies demonstrate that fungal diversity and network interactions in solid-state fermentation systems enhance the functional efficacy of fermentation processes. Such microbial complexity elevates fermented product quality through facilitation of carbon metabolic pathways and biosynthesis of flavor compounds. These findings strongly suggest that fungal communities play pivotal roles in shaping the functional enzyme repertoire and flavor characteristics of *Daqu* (Zhang, Xu, et al., 2022).

A critical question arises regarding how these low-abundance fungi maintain dominant enzymatic activities in high-temperature *Daqu*, where core temperatures typically exceed 60 °C—a threshold generally inhibitory to fungal growth. This phenomenon can be explained by a combination of biological, spatial, and temporal mechanisms. First, from a biological standpoint, certain endemic fungal populations in *Daqu* exhibit remarkable intrinsic thermotolerance, capable of surviving temperatures up to 55–60 °C (Zhou et al., 2022). These robust strains likely secrete thermotolerant isozymes that maintain catalytic stability even under high thermal stress. Second, the solid-state architecture of *Daqu* bricks creates highly heterogeneous spatial micro-niches(Yang, Fu, et al., 2024). While the core undergoes intense thermogenesis, the outer crust and peripheral layers experience significant heat dissipation, providing a cooler thermal refuge that permits fungal survival and active enzyme secretion. Third, and crucially from a temporal perspective, *Daqu* production involves a prolonged storage (maturation) phase following peak fermentation (M. Zhu, Zheng, et al., 2022). As the *Daqu* cools, it creates an environmental window that permits the resuscitation of heat-stressed fungi and the secondary colonization of ambient fungi from the storage environment. These newly introduced or recovered fungi can rapidly proliferate and secrete enzymes during storage, thereby contributing significantly to the accumulated functional proteome observed in mature *Daqu*.

In the upstream region, the abundances of *Oceanobacillus*, *Debaryomyces*, *Monascus*, *Saccharomonospora*, *Heyndrickxia*, and *Staphylococcus* were significantly higher. The midstream region exhibited higher abundances of *Rhizopus*, *Penicillium*, *Thermoactinomyces*, *Pallidibacillus*, *Rasamsonia*, *Saccharopolyspora*, and *Paecilomyces*. Downstream regions were dominated by *Hyphopichia*, *Pichia*, and *Weissella*. The spatial distribution of microbial protein abundances largely aligned with metagenomic data but showed notable differences. Specifically, proteomic data revealed lower protein abundances for *Kroppenstedtia*, *Lentibacillus*, and *Pallidibacillus*—dominant in metagenomes—whereas fungi like *Monascus*, *Penicillium*, and *Lichtheimia*, though prevalent in metagenomes, exhibited variable and non-dominant abundances in the proteome. Notably, *Hyphopichia*, nearly undetected in metagenomes, consistently dominated the proteomes of all *Daqu* samples, suggesting that low-abundance microorganisms may critically contribute to enzymatic functions. While this “abundance-activity paradox” underscores the potential of rare biosphere members acting as keystone functional executors, it is imperative to interpret this discrepancy with methodological caveats. The divergence between genomic potential and expressed protein profiles may not be exclusively biological; several technical biases inherent to multi-omics approaches could also contribute to this pattern. First, bias in DNA extraction efficiency is a well-documented challenge in microbiome research; thick-walled fungal spores or specific cellular structures of *Hyphopichia* might resist complete lysis during total DNA extraction, leading to a significant underestimation of its true genomic abundance (Luise R et al., 2025). Second, inherent biological variations, such as differences in rRNA gene copy numbers among diverse fungal species, can further skew taxonomic profiling and relative abundance calculations in sequencing data (Matchado et al., 2024). Third, the metagenomic assembly process inherently struggles with low-abundance taxa (Anwar et al., 2025). It is highly probable that the fragmented reads of *Hyphopichia* experienced “assembly dropouts,” failing to assemble into recognizable contigs due to insufficient sequencing depth for the rare biosphere. Conversely, from a metaproteomic perspective, the high sequence homology among related fungal proteins might lead to incorrect protein annotations or false-positive taxonomic assignments during database searching, artificially inflating the apparent functional dominance of *Hyphopichia* (Conti et al., 2023). Therefore, while our integrated omics approach strongly suggests that low-abundance taxa drive critical metabolic fluxes, acknowledging these technical limitations is crucial. Future in vitro validation—such as absolute quantification via qPCR or targeted parallel reaction monitoring proteomics—is required to definitively decouple actual biological activity from methodological artifacts. Principal component analysis (PCA) of *Daqu* proteins from upstream, midstream, and downstream sections of the Chishui River (Fig. 5B) revealed distinct separation, indicating functionally unique microbial communities in different river segments that may influence *Daqu* functionality. Hierarchical clustering analysis using Euclidean distance matrices and average linkage (Fig. 5C) grouped samples primarily by watershed, with downstream samples showing lower dispersion (*Daqu*_6 formed a separate cluster). We further analyzed microbial relative abundances across taxa (Fig. 5D). Results showed higher bacterial abundances (*Oceanobacillus*, *Staphylococcus*, *Bacillus*) upstream; increased fungal abundances (*Penicillium*, *Monascus*, *Aspergillus*, *Talaromyces*, *Rasamsonia*) alongside elevated bacteria (*Pallidibacillus*, *Thermoactinomyces*, *Saccharopolyspora*, *Streptomyces*) midstream; and higher abundances of *Hyphopichia*, *Weissella*, and *Aspergillus* downstream.

**Fig. 6:**
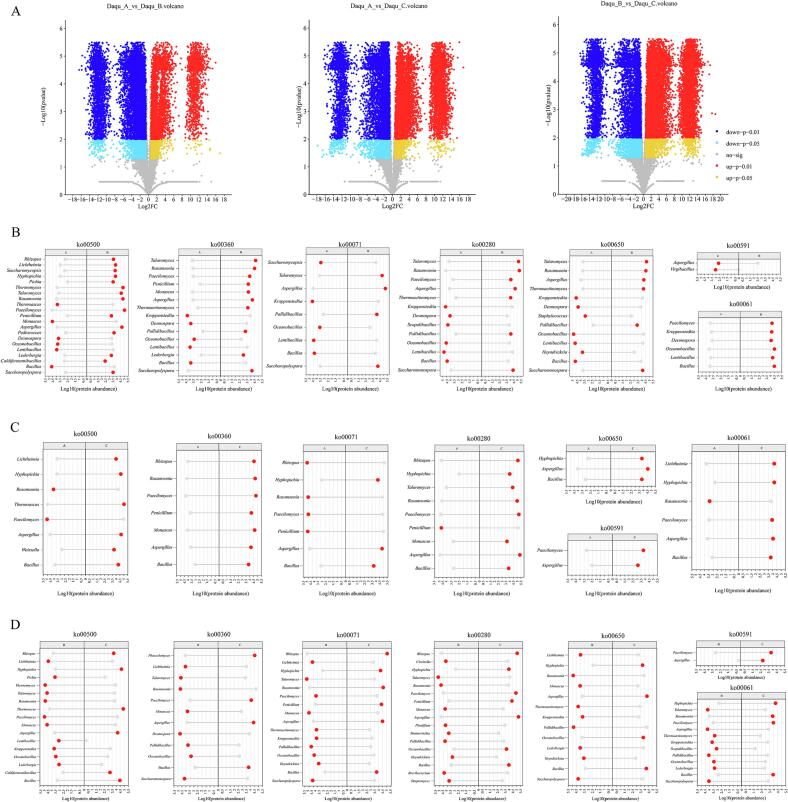
(A) Differentially expressed proteins in *Daqu* across distinct drainage basins. (B) Differentially abundant microorganisms within functional KEGG pathways for Group A versus Group B. (C) Differentially abundant microorganisms within functional KEGG pathways for Group B versus Group C. (D) Differentially abundant microorganisms within functional KEGG pathways for Group C versus Group D.

Furthermore, we quantified core and basin-specific microbial proteins in *Daqu* samples from distinct river watersheds. The three basins shared 25,945 proteins, with Group A exhibiting 434 differentially abundant proteins, while Groups B and C contained 1686 and 1230 unique proteins, respectively. Enzyme enrichment analysis based on KEGG annotations revealed *Daqu*'s functional signatures linked to volatile compound profiles. Within the shared enzymatic repertoire, pyruvate metabolism represented the most dominant pathway (45 enzymes), succeeded by glycine, serine and threonine metabolism (42 enzymes), amino sugar and nucleotide sugar metabolism (39 enzymes), cysteine and methionine metabolism (34 enzymes), pentose and glucuronate interconversions (32 enzymes), glycolysis/gluconeogenesis (32 enzymes), and starch and sucrose metabolism (32 enzymes). Critically, carbohydrate- and amino acid-related pathways demonstrated cross-basin conservation. Differential enzyme analysis indicated that group-specific enzymatic markers consistently correlated with cysteine and methionine metabolism, glyoxylate and dicarboxylate metabolism, glycolysis/gluconeogenesis, and degradation pathways for valine, leucine, and isoleucine. In addition to the unique enzymes, we further analyzed the differences in shared proteins among different *Daqu* samples (Fig. 6A). Based on fold change (FC) and *p*-values, we compared the proteomic differences between *Daqu* from different drainage basins. Compared to Group B, Group A exhibited 7406 up-regulated proteins and 12,735 down-regulated proteins. Group A showed 10,567 up-regulated proteins and 12,846 down-regulated proteins versus Group C. Group B demonstrated 12,777 up-regulated and 9474 down-regulated proteins compared to Group C, further indicating functional expression disparities among *Daqu* from three distinct basins.

Using predefined thresholds (*p* < 0.01, FC ≥2 or ≤ 0.5), we screened KEGG pathways related to *Daqu* functions based on prior research(Luo, Liao, Wu, et al., 2025), and identified relevant enzymes and associated microbes. In carbohydrate metabolism pathways (ko00500), Group A showed significantly higher abundance of enzymes linked to *Thermoascus*, *Monascus*, *Desmospora*, *Oceanobacillus*, *Lentibacillus*, and *Bacillus* compared to Group B. Group B exhibited greater abundance of *Thermomyces*, *Talaromyces*, *Rasamsonia*, *Paecilomyces*, and *Aspergillus* (Fig. 6B). For Group C, *Lichtheimia*, *Hyphopichia*, *Weissella*, and *Bacillus* also participated in carbohydrate degradation, with abundances significantly higher than Group A. These differences may reflect selective pressure from differential substrate availability on microbiota across basins. Bacteria typically adapt better to rapidly degrade simple carbohydrates (e.g., glucose, maltose, sucrose)(Jiang et al., 2020),while fungi likely play key roles in decomposing complex polysaccharides like starch and cellulose (S.-B. Yang et al., 2025). This emphasizes the importance of microbial functional complementarity in maintaining ecosystem stability. From an ecological and evolutionary perspective, the ‘abundance-activity paradox’ observed in *Bacillus* highlights a classic temporal decoupling between biomass accumulation and metabolic activity (Bressuire-Isoard et al., 2018). During the initial stages of *Daqu* fermentation, when temperatures are moderate, *Bacillus* likely adopts an r-selection strategy, rapidly proliferating to occupy the ecological niche. However, as the core temperature peaks (>60 °C), these bacteria undergo sporulation to endure the severe environmental stress. Consequently, while metagenomic sequencing captures the massive genomic DNA from these resilient endospores, their actual protein synthesis is virtually halted, explaining their low in situ protein expression in the mature *Daqu*. Different microorganisms may optimize resource utilization and avoid competitive exclusion through division of labor. In the phenylalanine-derived aromatic volatile compound pathway (ko00360), the abundances of bacteria such as *Kroppenstedtia*, *Desmospora*, *Pallidibacillus*, *Oceanobacillus*, *Lentibacillus*, and *Bacillus* in Group A were significantly higher than those in Group B. In contrast, fungi including *Talaromyces*, *Rasamsonia*, *Paecilomyces*, and *Penicillium* in Group B exhibited greater enzyme expression. Furthermore, in Group C, except for *Phascolomyces*, *Paecilomyces*, *Aspergillus*, and *Bacillus*, the abundances of related enzymes in other microorganisms were lower than in Group B. This indicates that Group B has distinctive characteristics in aromatic amino acid metabolism, followed by Group C. Studies show that phenylalanine, as an aromatic amino acid, produces metabolites such as 2-phenylethanol (2-PE), which is highly valued for its rose-like aroma. Prior research revealed that phenylalanine supplementation during soy (tofu) whey fermentation significantly altered flavor profiles by promoting sugar utilization and enhancing the formation of higher alcohols and their corresponding esters. This further underscores the critical role of fungi in aromatic amino acid metabolism (Chua & Liu, 2020),consistent with our earlier detection of notably higher alcohol content in *Daqu* starter from the C watershed. In the long-chain volatile compound pathway (ko00071), the abundances of enzymes expressed by microorganisms like *Saccharomycopsis*, *Kroppenstedtia*, *Oceanobacillus*, and *Lentibacillus* in Group A were significantly higher than in Group B, while fungi such as *Rhizopus*, *Rasamsonia*, *Paecilomyces*, and *Penicillium* showed higher abundances than in Group C. Compared to Group B, Group C displayed elevated fungal enzyme expression, whereas Group B primarily exhibited bacterial enzyme expression. In the volatile compound pathway of branched-chain amino acids, Group A mainly shows high expression of bacteria-related enzymes, including *Kroppenstedtia*, *Desmospora*, *Oceanobacillus*, and *Lentibacillus*. Compared with Group A, Group B exhibits high expression of fungi-related enzymes such as *Rasamsonia*, *Paecilomyces*, and *Aspergillus*. Additionally, Group C also displays high expression of fungi-related enzymes relative to Group A, including *Rhizopus*, *Talaromyces*, and *Rasamsonia* (Fig. 6C). In the pyrazine synthesis pathway, acetoin is a critical precursor compound. Its metabolism involves the ko00650 pathway, where Group A expresses predominantly bacterial enzymes. The abundance of bacteria-related enzymes in Group B is significantly higher than in Group C (Fig. 6D). Unlike other groups, the expression of *Bacillus* enzymes is substantially higher in Groups A and C compared to Group B. Studies demonstrate that *Bacillus* can efficiently produce acetoin and further convert it to tetramethylpyrazine (TMP) (Peng et al., 2020). Our previous research revealed significantly higher 4-methylpyrazine content in Groups A and C than in Group B, indicating that enhanced *Bacillus*-related enzyme expression likely drives this outcome. In the fatty acid synthesis pathway (ko00061), Group B exhibited higher abundances of bacteria-associated enzymes such as *Kroppenstedtia*, *Thermoactinomyces*, *Scopulibacillus*, and *Oceanobacillus*, while Group C showed higher abundances of fungi-associated enzymes such as *Hyphopichia*, *Rasamsonia*, and *Paecilomyces*. For volatile compounds derived from long-chain fatty acids (ko00591), Group C demonstrated significantly higher enzyme abundances of microbes including *Aspergillus* and *Paecilomyces* than other groups, indicating these compounds are primarily mediated by fungi. Previous studies employing high-throughput amplicon sequencing combined with metagenomic and metatranscriptomic analyses identified *Kroppenstedtia* as an important contributor to fatty acid synthesis during fermentation of Chinese soy-sauce aroma-type baijiu. This finding highlights the significance of *Kroppenstedtia* in fatty acid synthesis (J. Zhang et al., 2024). Furthermore, members of *Actinobacteria* possess diverse fatty acid synthesis capabilities, exemplified by the FAS II system in *Streptomyces*(Gago et al., 2011), though the fatty acid synthesis system in *Thermoactinomyces* warrants further investigation. These patterns strongly suggest functional niche partitioning of microbial communities under varying environmental or treatment conditions.

### 3.6 Metabolic network underlying core HTD metabolism

**Fig. 7:**
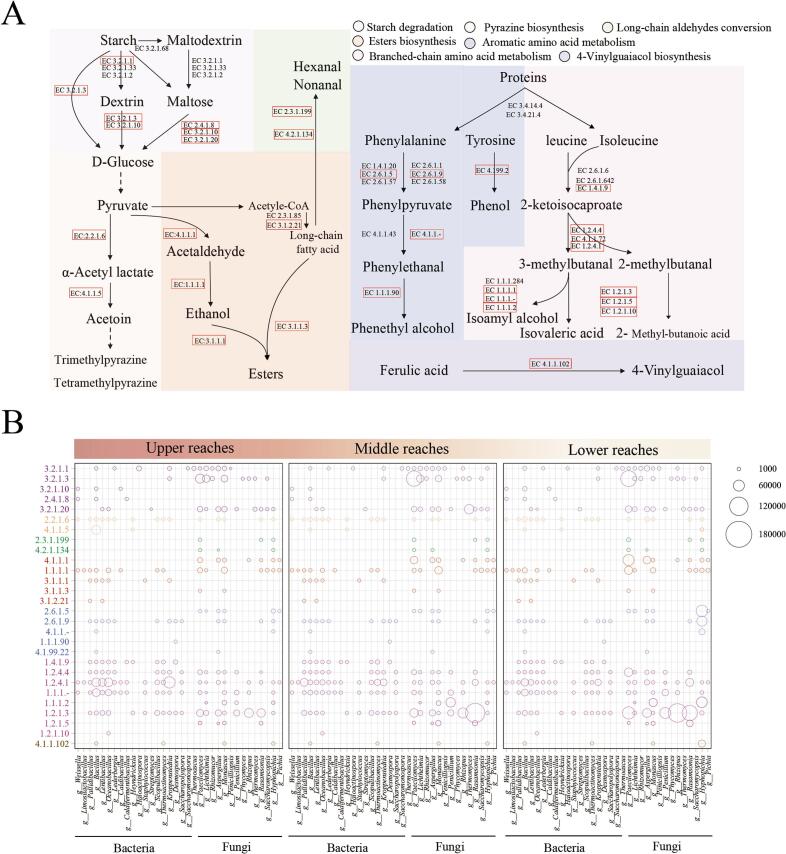
(A) The organization of the high-temperature *Daqu* metabolic network comprising seven core modules; (B) Spatial distribution of microbiota and enzymatic profiles associated with substrate catabolism and flavor-biosynthetic pathways within the *Daqu* ecosystem.

To determine the expression levels of enzyme-coding genes critical for substrate degradation and characteristic flavor formation, and to assess the contributions of core microorganisms, we reconstructed a metabolic network encompassing seven key pathways based on KEGG database annotations (Fig. S2). We identified 42 relevant enzymes, and their protein abundance profiles are presented in the heatmap (Fig. 7A). Given the complexity and diversity of starch saccharification routes, we evaluated enzymes across multiple pathways to pinpoint the principal routes for starch decomposition in *Daqu*. Key enzyme abundances within the starch decomposition module were substantially higher in B and C samples compared to the A. This finding aligns with the lower saccharification power observed in A. The starch breakdown pathways primarily involved conversion to glucose via intermediate steps like dextrin and maltose. Phenylalanine degradation largely involved EC 2.6.1.5 and EC 2.6.1.9, while branched-chain amino acid degradation was chiefly mediated by EC 1.4.1.9. Starch degradation capacity and flavor compound production are paramount for evaluating HTD quality. These processes integrate substrate degradation with precursor biosynthesis, driven by synergistic microbial metabolism. Liquefaction and saccharification powers measure microbial starch degradation efficiency and function by swelling and rupturing starch granules, creating a homogeneous paste to enhance subsequent saccharification. Starch-to-glucose conversion primarily occurs via maltose alpha-D-glucosyltransferase/alpha-amylase (EC 3.2.1.1), producing dextrin and maltose intermediates, or directly via EC 3.2.1.3. Notably, EC 3.2.1.3 was predominantly produced by fungi (*Paecilomyces*, *Lichtheimia*, *Monascus*, *Rhizomucor*, *Thermomyces*, *Saccharomycopsis*) (Fig. 7C), although some bacteria (*Bacillus*, *Kroppenstedtia*, *Lederbergia*) also contributed. Bacteria like *Weissella* and *Bacillus* possessed enzymes targeting maltose and dextrin (e.g., EC 3.2.1.10, EC 2.4.1.8). Consequently, *Bacillus* and *Kroppenstedtia* likely obtained carbon directly from starch depolymerization, whereas bacteria like *Weissella* appeared dependent on fermentable sugars (maltose, glucose) supplied by others, suggesting microbial community assembly in *Daqu* is influenced by substrate selectivity. Crucially, within B and C, the significantly higher abundance of EC 3.2.1.3 attributed to *Paecilomyces* likely underpinned their enhanced saccharification power compared to A. Tetramethylpyrazine is generated through a highly efficient bio-chemo synergistic mechanism rather than a strictly enzymatic pathway. Upstream *Daqu* exhibited elevated abundances of bacterial acetolactate decarboxylase (predominantly from *Bacillus*), which is crucial for the massive biological accumulation of the precursor, acetoin (*P.* wang et al., 2017). However, from a food chemistry perspective, the final conversion of acetoin to TMP is primarily driven by the abiotic, thermally-induced Maillard reaction, naturally facilitated by the >60 °C peak temperature (H. Xu et al., 2024). Thus, TMP enrichment is the cumulative result of biological precursor supply coupled with optimal abiotic thermal conditions. This correlates with the higher pyrazine content found in A. Long-chain fatty aldehyde synthesis involved enzymes like EC 2.1.3.199, predominantly expressed by fungi (*Paecilomyces*, *Hyphopichia*). For ester synthesis, short-chain esters depended mainly on carboxylesterase (EC 3.1.1.1) from bacteria (*Pallidibacillus*, *Bacillus*), while long-chain esters relied on triacylglycerol lipase (EC 3.1.1.3) from fungi (*Paecilomyces*, *Monascus*), Research has shown that Monascus purpureus can synthesize short-chain fatty acid esters in aqueous phases, and our findings are consistent with this(Y. Xu et al., 2021). Aromatic amino acid degradation involved EC 2.6.1.5 (expressed by fungi like *Thermoascus*, *Pichia*) and EC 2.6.1.9 (expressed by bacteria like *Bacillus*, *Pallidibacillus*, *Oceanobacillus*). Aryl-alcohol dehydrogenase (EC 1.1.1.90) was uniquely expressed by *Desmospora*. However, given the high abundance and overlapping function of enzymes like EC 1.1.1.- or EC 1.1.1.2 in HTD, these likely contribute to the synthesis of aromatic alcohols (e.g., phenethyl alcohol) and were expressed mainly by fungi (*Hyphopichia*, *Monascus*, *Lichtheimia*). BCAAs were degraded primarily via leucine dehydrogenase (EC 1.4.1.9) expressed by bacteria (*Pallidibacillus*, *Lentibacillus*, *Bacillus*), with subsequent conversion of aldehydes to acids (e.g., isovaleric acid) by aldehyde dehydrogenases (EC 1.2.1.3, EC 1.2.1.5, EC 1.2.1.10), the importance of branched-chain amino acids in microbial metabolism can be understood through their role in energy metabolism. Branched-chain amino acids serve as crucial energy sources, particularly in the absence of glucose. Research indicates that branched-chain amino acids can be converted into acetyl-CoA and enter the tricarboxylic acid cycle, thereby supplying energy to cells (Chen et al., 2024). Although bacteria such as *Pallidibacillus*, *Lentibacillus*, and *Bacillus* can also encode these aldehyde dehydrogenases, they exhibit high expression in fungi (*Rasamsonia*, *Thermomyces*, *Rhizopus*) and even higher expression in B and C, indicating fungi as key producers of characteristic branched-chain acids like isovaleric acid. Similarly, the biosynthesis of 4-vinylguaiacol is associated with *Bacillus*, *Thermoactinomyces*, *Hyphopichia*, and *Monascus*. Among these, *Hyphopichia* shows significantly higher abundance of ferulic acid decarboxylase (EC 4.1.1.102), particularly enriched in C. This finding aligns with the peak levels of 4-vinyl guaiacol detected in downstream *Daqu*. Numerous studies have demonstrated that microorganisms including *Bacillus*, *Enterobacter*, *Streptomyces*, and *Lactobacillus* convert ferulic acid to 4-vinylguaiacol via ferulic acid decarboxylase (EC 4.1.102)(Hadiza et al., 2012; Hunter et al., 2012; L.-H. Sun et al., 2018). Nevertheless, the microbial origins of guaiacol derivatives synthesis in (HTD) remain elusive. We plan to experimentally verify the key microorganisms contributing to guaiacol production in subsequent studies. Furthermore, our co-occurrence networks reveal a robust ‘cross-feeding’ mechanism. The high-abundance bacteria (such as *Bacillus*) function as pioneer degraders during early fermentation stages. By secreting robust extracellular proteases and amylases, they depolymerize recalcitrant macromolecules into bioavailable small peptides and fermentable sugars. This early-stage mass degradation paves the way for the later-stage fungi, supplying them with the essential small-molecule precursors required to drive the ultimate synthesis of complex flavor compounds (S. Liu et al., 2024). It is important to note that *Daqu* fermentation is a highly dynamic process lasting over a month. The current study relies on a single-time-point multi-omics snapshot of the mature *Daqu* products. While this effectively captures the cumulative outcomes and final active microbiota, it may introduce bias regarding the temporal succession of the community. Future studies employing time-series-based multi-omics sampling are warranted to fully map the dynamic cross-feeding networks and temporal shifts in gene expression.

## 4 Conclusion

This integrated multi-omics study systematically analyzed high-temperature *Daqu* starters across the Chishui River, elucidating the mechanistic basis for geographic heterogeneity in Maotai-flavor baijiu terroir. Geographic segregation drove distinct microbial community assembly: Upstream sites were dominated by thermotolerant *Kroppenstedtia* and *Saccharopolyspora*, concomitant with elevated pyrazine biosynthesis; whereas downstream proliferation of *Weissella* and *Hyphopichia* promoted alcohol generation. Crucially, metaproteomics confirmed that metabolic functional divergence was governed by in situ enzyme activities—starch saccharification was primarily mediated by *Paecilomyces* and *Hyphopichia* derived α-amylase, while upstream pyrazine accumulation was regulated by *Bacillus*-originated acetolactate decarboxylas. Although spatial distance directly diminished microbial taxonomic similarity, fungal co-occurrence networks sustained ecosystem stability. The disconnect between phylogenetic biomarkers and functional drivers underscores the necessity of activity-based microbial profiling for terroir prediction. These results establish robust microbiome-function-flavor linkages, offering industrial standardization biomarkers while preserving regional distinctiveness. Future work should prioritize in vitro validation of enzymatic pathways and practical implementation of synthetic microbial consortia. Collectively, our findings advocate for a paradigm shift in the assessment and engineering of fermentation starters: from a taxonomy-centric to a function- and activity-centric perspective. The identified functional niches and active microbial consortia provide a actionable roadmap for the rational design of synthetic communities, aiming to achieve both flavor standardization and the preservation of coveted geographical identities in fermented food production.

## CRediT authorship contribution statement

**Dandan Song:** Writing – review & editing, Writing – original draft, Validation, Funding acquisition, Formal analysis, Data curation, Conceptualization. **Xian Zhong:** Writing – review & editing, Validation, Formal analysis. **Guihu Zhang:** Writing – review & editing. **Juan Chen:** Writing – review & editing. **Yansong Xue:** Writing – review & editing, Conceptualization. **Liang Yang:** Writing – review & editing, Project administration, Investigation, Funding acquisition, Formal analysis, Data curation, Conceptualization.

## Declaration of competing interest

The authors declare that they have no known competing financial interests or personal relationships that could have appeared to influence the work reported in this paper.

## Data Availability

Data will be made available on request.
