# Supplementary material for: Linking geographic flavor signatures to microbial origin in high-temperature Daqu: An integrated metaproteomics and metabolomics approach
Source: Food Chem X. 2026 May 6;36:103952. doi: 10.1016/j.fochx.2026.103952 (PMC13188119; doi:10.1016/j.fochx.2026.103952)
Supplement: Supplementary file 1 — Supplementary material [file mmc1.docx]

**Supporting information for this manuscript**

**Table S1. Identification and differential abundance of non-volatile metabolites in high-temperature *Daqu* across distinct geographic regions within the Chishui River Basin.**

| sample | VIP | Class I | Class II | Molecular weight (Da) | RT (min) | Adduct | Fold change | | |
| --- | --- | --- | --- | --- | --- | --- | --- | --- | --- |
|  |  |  |  |  |  |  | A VS B | A VS C | B VS C |
| Isocitric acid | 6.73100668 | Organic acid and Its derivatives | Organic acid and Its derivatives | 192.027 | 0.8264 | [M-H]- | 0.35 | 0.30 | 0.86 |
| N-Acetylleucine | 5.016791174 | Amino acid and Its metabolites | Amino acid derivatives | 173.1052 | 3.3954 | [M-H]- | 2.45 | 1.38 | 0.56 |
| 2-Methylbenzoic acid | 4.712989027 | Benzene and substituted derivatives | Phenolic acids | 136.0524 | 4.3898 | [M-H]- | 2.20 | 1.95 | 0.89 |
| N-Acetylvaline | 4.331302182 | Amino acid and Its metabolites | Amino acid derivatives | 159.0895 | 2.5174 | [M-H]- | 3.79 | 2.13 | 0.56 |
| 4-Acetamidobenzoic acid | 3.61056512 | Benzene and substituted derivatives | Benzene and substituted derivatives | 179.0582 | 3.8319 | [M-H]- | 2.03 | 7.41 | 3.65 |
| L-Serine | 3.15198717 | Amino acid and Its metabolites | Amino acids | 105.0426 | 0.7893 | [M-H]- | 2.27 | 0.94 | 0.41 |
| Azelaic acid | 3.143891243 | Organic acid and Its derivatives | Organic acid and Its derivatives | 188.1049 | 4.1921 | [M-H]- | 0.82 | 1.67 | 2.04 |
| 4-Hydroxybenzaldehyde | 3.067535656 | Benzene and substituted derivatives | Benzene and substituted derivatives | 122.0368 | 3.5166 | [M-H]- | 0.58 | 1.41 | 2.42 |
| 3-(3-Hydroxyphenyl)Propionate Acid | 3.025008453 | Organic acid and Its derivatives | Organic acid and Its derivatives | 166.063 | 3.657 | [M-H2O-H]- | 2.32 | 1.41 | 0.61 |
| aldehydo-D-ribose | 2.904481005 | Carbohydrates and Its metabolites | Sugars | 150.0528 | 1.0261 | [M+HCOO]- | 0.63 | 1.41 | 2.23 |
| L-Valine | 2.449781553 | Amino acid and Its metabolites | Amino acids | 117.079 | 1.817 | [M-H]- | 2.09 | 0.80 | 0.38 |
| Ectoine | 2.421220085 | Organic acid and Its derivatives | Organic acid and Its derivatives | 142.0742 | 0.8195 | [M+H]+ | 1.96 | 2.20 | 1.12 |
| Ile-Asp | 2.330841847 | Amino acid and Its metabolites | Small Peptide | 246.1216 | 1.898 | [M+H-H2O]+ | 8.83 | 1.51 | 0.17 |
| Vanillin | 2.324109519 | Benzene and substituted derivatives | Phenolics | 152.0473 | 3.7699 | [M-H]- | 0.59 | 2.10 | 3.53 |
| N-Phenylacetylphenylalanine | 2.198117509 | Amino acid and Its metabolites | Amino acids | 283.1208 | 5.2755 | [M-H]- | 3.09 | 1.73 | 0.56 |
| Ethyl salicylate | 2.070317205 | Benzene and substituted derivatives | Benzene and substituted derivatives | 166.063 | 3.9685 | [M-H]- | 2.42 | 1.71 | 0.71 |
| N-Acetylneuraminic Acid(SA) | 2.052375124 | Organic acid and Its derivatives | Organic acid and Its derivatives | 309.106 | 0.8264 | [M-H]- | 0.65 | 0.34 | 0.53 |
| 2-Aminobenzoic acid | 1.987547381 | Phenolic acids | Phenolic acids | 137.0477 | 3.8319 | [M-H]- | 0.86 | 7.26 | 8.40 |
| δ-Valerolactam | 1.948163583 | Alcohol and amines | Amines | 99.0684 | 1.9722 | [M+H]+ | 2.16 | 3.31 | 1.54 |
| Hydrocinnamic acid | 1.919671255 | Organic acid and Its derivatives | Organic acid and Its derivatives | 150.0681 | 4.9497 | [M-H]- | 9.60 | 3.20 | 0.33 |
| Phe-Pro | 1.867803445 | Amino acid and Its metabolites | Amino acids | 262.1317 | 2.7339 | [M+H]+ | 2.23 | 0.56 | 0.25 |
| Succinylacetone | 1.839132418 | Organic acid and Its derivatives | Organic acid and Its derivatives | 158.0579 | 2.8085 | [M-H]- | 2.13 | 2.25 | 1.06 |
| 2-Phenylethanol | 1.779367082 | Benzene and substituted derivatives | Benzene and substituted derivatives | 122.0732 | 2.2355 | [M+H-H2O]+ | 3.00 | 1.95 | 0.65 |
| Uric acid | 1.622737806 | Organic acid and Its derivatives | Organic acid and Its derivatives | 168.0283 | 1.2125 | [M-H]- | 3.97 | 14.41 | 3.63 |
| Quinolinic acid | 1.61851604 | Organic acid and Its derivatives | Organic acid and Its derivatives | 167.0219 | 0.8284 | [M-]- | 4.30 | 17.21 | 4.00 |
| DL-3-Phenyllactic acid | 1.590640859 | Organic acid and Its derivatives | Organic acid and Its derivatives | 166.063 | 3.6509 | [M-H]- | 3.72 | 2.27 | 0.61 |
| Glycyl-L-leucine | 1.533360956 | Amino acid and Its metabolites | Amino acid derivatives | 188.1161 | 1.7485 | [M+H]+ | 1.01 | 0.20 | 0.20 |
| Diethyl hydrogen phosphate | 1.499563907 | Aldehyde,Ketones,Esters | Esters | 154.0395 | 0.8264 | [M+HCOO]- | 0.46 | 0.35 | 0.76 |
| 2-Phenoxyethanol | 1.475568483 | Alcohol and amines | Alcohols | 138.0681 | 1.2529 | [M+H-H2O]+ | 7.28 | 3.25 | 0.45 |
| Etofylline | 1.461752121 | Nucleotide and Its metabolites | Nucleotide and Its metabolites | 224.0909 | 1.2159 | [M+]+ | 0.75 | 0.39 | 0.52 |
| 2,2-Dimethylsuccinic acid | 1.445036294 | Organic acid and Its derivatives | Organic acid and Its derivatives | 146.0579 | 2.3871 | [M-H]- | 1.22 | 2.71 | 2.22 |
| Morin | 1.427562843 | Flavonoids | Flavonols | 302.0427 | 4.7377 | [M-H]- | 9.22 | 3.51 | 0.38 |
| Isoleucylleucine | 1.34794539 | Amino acid and Its metabolites | Small Peptide | 244.1787 | 2.7277 | [M+H]+ | 1.28 | 0.64 | 0.50 |
| 2-Methoxy-3,5-dimethylpyrimidine | 1.346432702 | Heterocyclic compounds | Heterocyclic compounds | 138.0793 | 2.4747 | [M+H]+ | 4.55 | 2.33 | 0.51 |
| Salsolinol | 1.338698868 | Heterocyclic compounds | Heterocyclic compounds | 179.0946 | 1.2094 | [M+H]+ | 10.02 | 3.77 | 0.38 |
| 4-Methylhippuric acid | 1.328366244 | Benzene and substituted derivatives | Benzene and substituted derivatives | 193.0739 | 4.4524 | [M-H]- | 1.28 | 6.31 | 4.91 |
| N-Heptanoylglycine | 1.308805882 | Amino acid and Its metabolites | Amino acid derivatives | 187.1208 | 4.3024 | [M-H]- | 5.26 | 4.01 | 0.76 |
| N-acetylornithine | 1.307709736 | Amino acid and Its metabolites | Amino acid derivatives | 174.1004 | 0.8318 | [M+H-H2O]+ | 2.01 | 1.75 | 0.87 |
| Metanephrine | 1.249042057 | Hormones and hormone related compounds | Hormones and hormone related compounds | 197.1052 | 1.3522 | [M+NH4]+ | 1.11 | 0.52 | 0.47 |
| Tyr-Pro | 1.211001359 | Amino acid and Its metabolites | Small Peptide | 278.1267 | 1.9351 | [M+H]+ | 1.13 | 0.43 | 0.38 |
| N-Acetylarylamine | 1.205311543 | Benzene and substituted derivatives | Benzene and substituted derivatives | 135.0684 | 3.8319 | [M-H]- | 2.00 | 7.19 | 3.60 |
| Tiglic acid | 1.190690388 | Organic acid and Its derivatives | Organic acid and Its derivatives | 100.0524 | 2.4677 | [M-H]- | 6.57 | 6.33 | 0.96 |
| L-Allothreonine | 1.18733185 | Amino acid and Its metabolites | Amino acids | 119.0582 | 0.0929 | [M+H-2H2O]+ | 0.91 | 0.50 | 0.55 |
| 2,3-Dihydrobenzofuran | 1.184724499 | Benzene and substituted derivatives | Benzene and substituted derivatives | 120.0575 | 1.7985 | [M+H]+ | 5.50 | 3.23 | 0.59 |
| Glutamine | 1.163696812 | Amino acid and Its metabolites | Amino acids | 146.0691 | 0.8034 | [M+H]+ | 1.41 | 0.32 | 0.23 |
| 3-Amino-4-phenylbutanoic acid | 1.146653439 | Amino acid and Its metabolites | Amino acids | 179.0946 | 1.9783 | [M+NH4]+ | 8.45 | 2.68 | 0.32 |
| 2,3,5,6-Tetramethylpyrazine | 1.133217046 | Heterocyclic compounds | Heterocyclic compounds | 136.1 | 4.2366 | [M+H]+ | 26.78 | 2.40 | 0.09 |
| Proline betaine | 1.122894306 | Amino acid and Its metabolites | Amino acid derivatives | 143.0946 | 0.8195 | [M+H]+ | 1.98 | 2.39 | 1.21 |
| Kainic acid | 1.102696021 | Organic acid and Its derivatives | Organic acid and Its derivatives | 213.1001 | 1.7982 | [M+NH4]+ | 1.88 | 2.46 | 1.31 |
| Hydroxyquinoline | 1.089074115 | Heterocyclic compounds | Heterocyclic compounds | 145.0528 | 2.6028 | [M+H]+ | 1.21 | 2.06 | 1.70 |
| Myosmine | 1.042011849 | Heterocyclic compounds | Heterocyclic compounds | 146.0844 | 2.1106 | [M+H]+ | 3.97 | 1.94 | 0.49 |
| 2-Phenylbutyramide | 1.037851535 | Benzene and substituted derivatives | Benzene and substituted derivatives | 163.0997 | 2.0605 | [M+NH4]+ | 31.22 | 6.81 | 0.22 |
| Aceclidine | 1.020041 | Heterocyclic compounds | Heterocyclic compounds | 169.1103 | 3.2399 | [M+H]+ | 23.00 | 10.63 | 0.46 |

Note: A: Upstream region of the Chishui River; B: Midstream region; C: Downstream region. VIP: Variable Importance in Projection, derived from the PLS-DA model. Metabolites with VIP > 1 are considered primary contributors to group separation. RT: Retention Time in liquid chromatography. Da: Daltons (molecular weight). Adduct: Ionization adduct formed during LC-MS analysis. Fold change: The ratio of the mean metabolite concentration between two comparative groups (e.g., A VS B). Fold change values have been rounded to two decimal places. p-value: Statistical significance of the differential abundance *calculated via T – test ,*and all metabolites listed in this table represent significantly differential compounds that have been strictly pre-filtered with a statistical significance threshold of *p < 0.05.*.


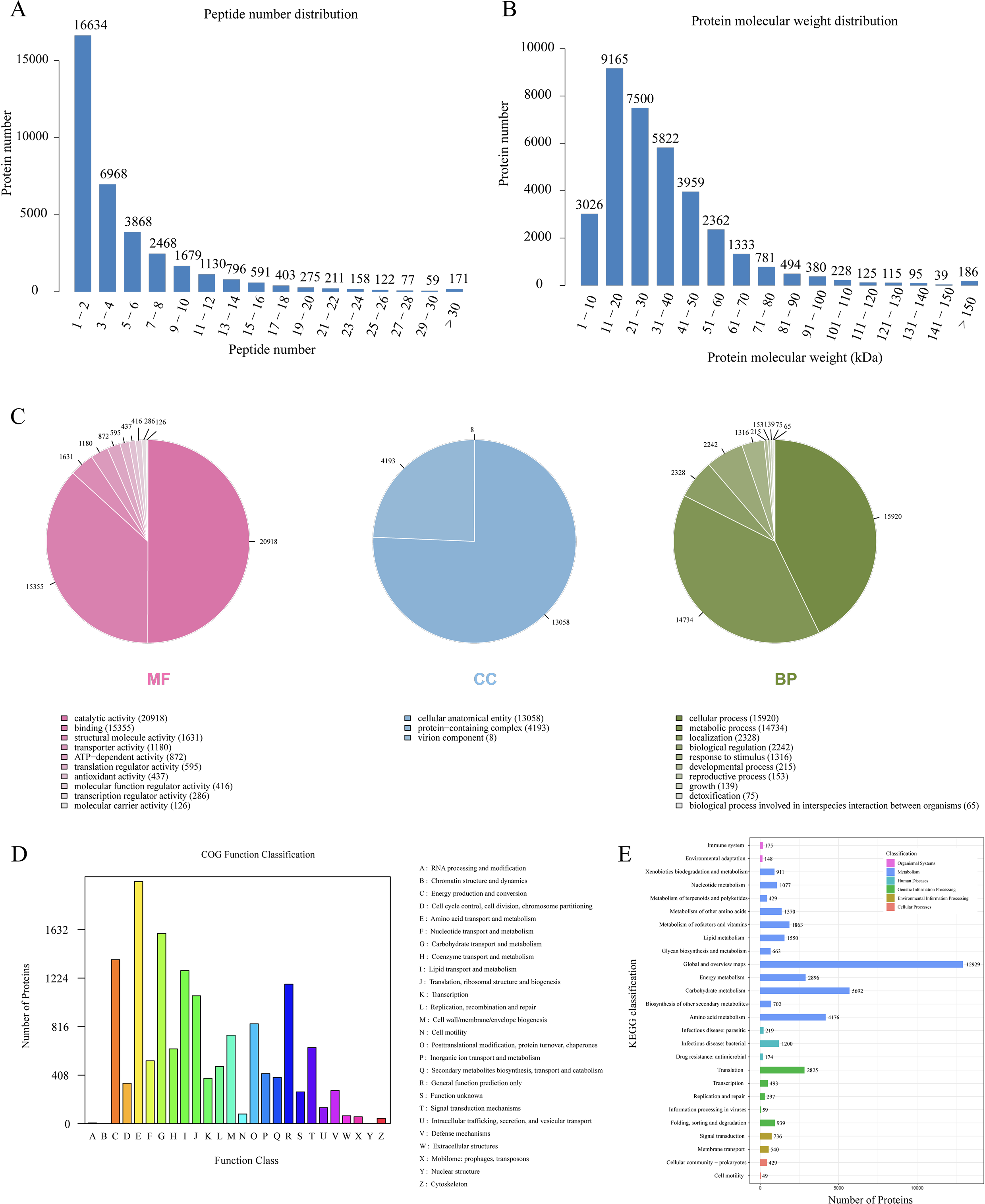


**Figure S1. The COG and KEGG annotation of protein functional classification**. (A) Number distribution of proteins; (B) Length distribution of proteins; (C) Functional annotation of proteins; (D) COG classification of proteins; (E) KEGG classification of proteins.


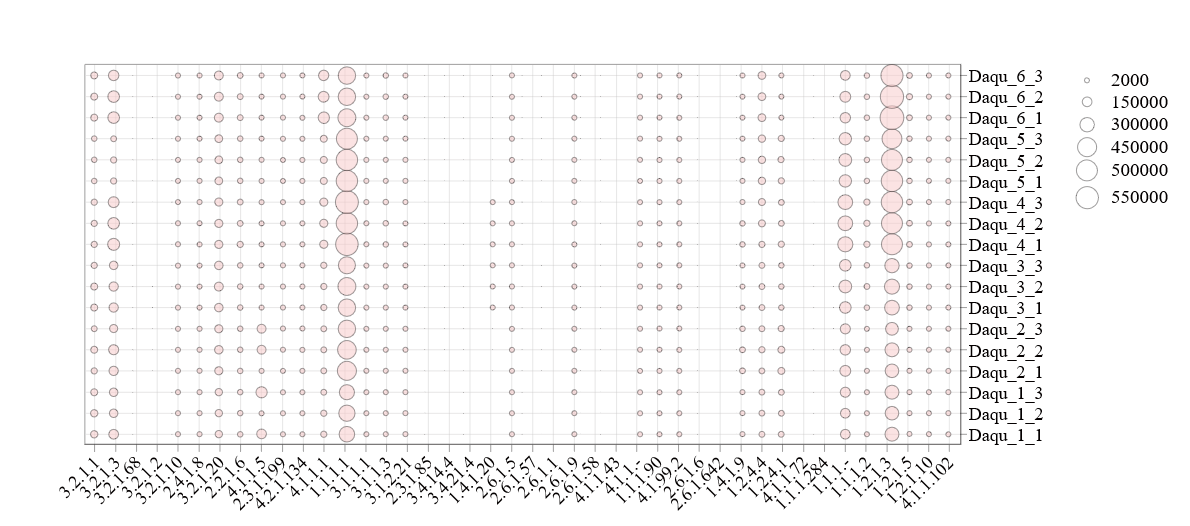


**Figure S2. Compartment-specific profiles of functional enzymes across distinct *Daqu* types.**
